# Temperature dependence of electronic behaviors in quantum dimension junctionless thin-film transistor

**DOI:** 10.1186/1556-276X-9-392

**Published:** 2014-08-13

**Authors:** Ya-Chi Cheng, Hung-Bin Chen, Ming-Hung Han, Nan-Heng Lu, Jun-Ji Su, Chi-Shen Shao, Yung-Chun Wu

**Affiliations:** 1 Department of Engineering and System Science, National Tsing Hua University, Section 2 Kuang Fu Road, Hsinchu 30013, Taiwan; 2 Department of Electronics Engineering & Institute of Electronics, National Chiao Tung University, 1001, Ta Hsueh Road, Hsinchu 30013, Taiwan

**Keywords:** Junctionless, Nanowire, Thin-film transistor (TFTs), Gate-all-around (GAA), Quantum confinement effect

## Abstract

The high temperature dependence of junctionless (JL) gate-all-around (GAA) poly-Si thin-film transistors (TFTs) with 2-nm-thick nanosheet channel is compared with that of JL planar TFTs. The variation of SS with temperature for JL GAA TFTs is close to the theoretical value (0.2 mV/dec/K), owing to the oxidation process to form a 2-nm-thick channel. The bandgap of 1.35 eV in JL GAA TFTs by fitting experimental data exhibits the quantum confinement effect, indicating greater suppression of I_off_ than that in JL planar TFTs. The measured $\frac{\partial V_{\mathrm{th}}}{\partial T}$ of −1.34 mV/°C in JL GAA nanosheet TFTs has smaller temperature dependence than that of −5.01 mV/°C in JL planar TFTs.

## Background

The junctionless nanowire transistor (JNT), which contains a single doping species at the same level in its source, drain, and channel, has been recently investigated [1-6]. The junctionless (JL) device is basically a gated resistor, in which the advantages of junctionless devices include (1) avoidance of the use of an ultra shallow source/drain junction, which greatly simplifies the process flow; (2) low thermal budgets owing to implant activation anneal after gate stack formation is eliminated, and (3) the current transport is in the bulk of the semiconductor, which reduces the impact of imperfect semiconductor/insulator interfaces. As is widely recognized, the temperature dependence of threshold voltage (*V*_th_) is a parameter when integrated circuits often operate at an elevated temperature owing to heat generation. This effect, accompanied with the degradation of subthreshold swing (SS) with temperature, causes the fatal logic errors, leakage current, and excessive power dissipation. Despite a previous work that characterized JNTs at high temperatures [7], there is no information regarding the JL thin-film transistor (TFT) at a high temperature yet. Hence, this letter presents a high-temperature operation of JL TFTs with a gate-all-around structure (GAA) for an ultra-thin channel. The JL TFT with a planar structure functions as the control device. The drain current (*I*_d_), SS, off-leakage current (*I*_off_), and *V*_th_ are also evaluated for fabricated devices. The JL GAA TFTs with a small variation in temperature performances along with simple fabrication are highly promising for future system-on-panel (SOP) and system-on-chip (SOC) applications.

## Methods

The process for producing 2-nm-thick poly-Si nanosheet channel was fabricated by initially growing a 400-nm-thick thermal silicon dioxide layer on 6-inch silicon wafers. Subsequently, a 40-nm-thick undoped amorphous silicon (a-Si) layer was deposited by low-pressure chemical vapor deposition (LPCVD) at 550°C. Then, the a-Si layer was solid-phase recrystallized (SPC) and formed large grain sizes as a channel layer at 600°C for 24 h in nitrogen ambient. The channel layer was implanted with 16-keV phosphorous ions at a dose of 1 × 10^14^ cm^−2^, followed by furnace annealing at 600°C for 4 h. Subsequently, we performed a wet trimming process with a dilute HF chemical solution at room temperature and shrink down channel thickness to be around 28 nm. The active layers, serving as channel, were defined by e-beam lithography and then mesa-etched by time-controlled wet etching of the buried oxide to release the poly-Si bodies. Subsequently, a 13-nm-thick dry oxide, consuming around 13-nm-thick poly-Si on both side of channel to form 2-nm-thick channel, and 6-nm-thick nitride by LPCVD were deposited as the gate oxide layer. The 250-nm-thick in-situ doped n + poly-silicon was deposited as a gate electrode, and patterned by e-beam and reactive ion etching. Finally, passivation layer and metallization was performed. The JL planar TFT serves as a control with single gate structure.

## Results and discussion

**Figure 1:**
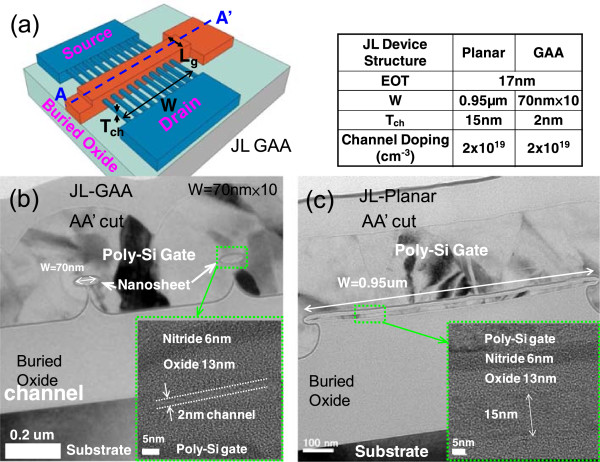
**JL GAA device structure in JL TFTs and TEM images for JL GAA and JL planar. (a)** The JL GAA device structure and relevant parameters in JL TFTs. The positions A and A′ indicate cross section of channel. **(b,c)** The TEM images along AA′ direction for JL GAA and JL planar with 2- and 15-nm channel thickness, respectively.

**Figure 2:**
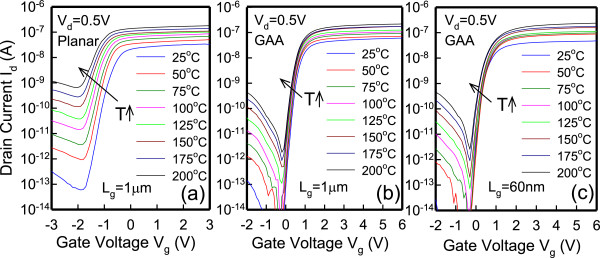
**Temperature dependence (25°C to 200°C) on *I***_**d **_**– *V***_**g **_**characteristics at *V***_**d**_ **= 0.5 V.** For JL GAA TFTs (*L*_g_ = 1 μm **(b)**, 60 nm **(c)**) and JL planar TFTs (*L*_g_ = 1 μm **(a)**). The *V*_th_ decreases and the SS increases with increasing temperature in both device structures.

**Figure 3:**
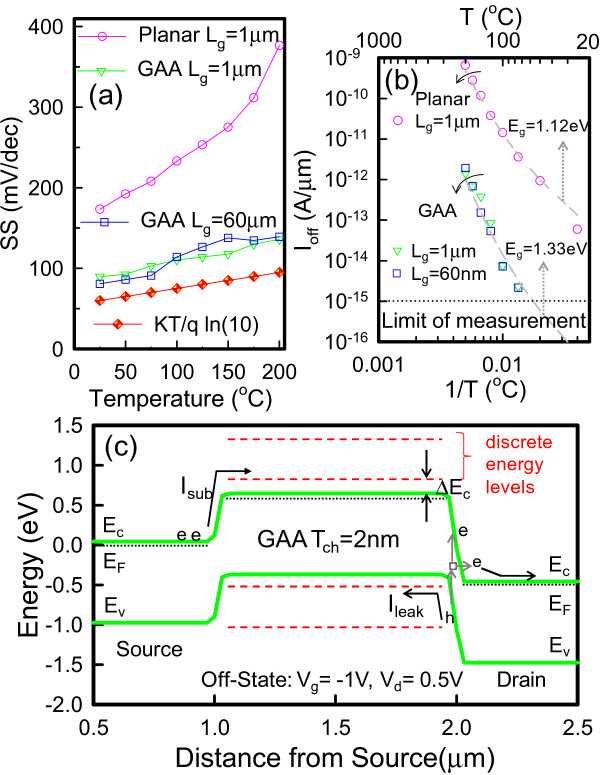
**Measured SS and *I***_**off **_**as function of temperature (a,b) and simulated band diagram of GAA structure (c). (a,b)** At *V*_d_ = 0.5 V, extracted from the *I*_d_ – *V*_g_ curves in Figure 2. **(c)** In the off-state with discrete energy levels and the Δ*E*_c_ is estimated around 0.23 eV.

Figure 1a presents the structure of the devices and relevant experimental parameters. Figure 1b displays the cross sectional transmission electron microscopic (TEM) images along the AA′ direction in JL GAA devices with ten strips of nanosheet; the figure clearly shows that the 2-nm-thick nanosheet channel is surrounded by the gate electrode. The dimensions of each nanosheet are 2-nm high × 70-nm wide. Figure 1c displays the TEM images in JL planar devices, and the channel dimensions are 15-nm high × 0.95-μm wide. Figure 2 shows the measured *I*_d_ as a function of gate bias (*V*_g_) at various temperatures ranging from 25°C to 200°C at *V*_d_ = 0.5 V for (a) JL planar TFTs with channel length (*L*_g_) of 1 μm, (b) JL GAA TFTs with *L*_g_ = 1 μm, and (c) JL GAA TFTs with *L*_g_ = 60 nm. This figure reveals that *V*_th_ decreases and the SS increases in all devices when increasing the temperature. Figure 3 presents the measured SS and *I*_off_ as a function of temperature at *V*_d_ = 0.5 V, as extracted from the *I*_d_-*V*_g_ curves in Figure 2. In Figure 3a, the JL GAA TFTs have a small SS variation with temperature than JL planar TFTs. Furthermore, the SS can be expressed as follows [8]:

(1) $$\mathrm{SS}=\left(\frac{\mathrm{kT}}{q}\right)\ln10\left(1+\frac{q^{2}t_{\mathrm{Si}}N_{T}}{C_{\mathrm{ox}}}\right),$$

where kT is the thermal energy, *C*_ox_ is the gate oxide capacitance per unit area, *N*_T_ is the trap states, and *t*_Si_ is the thickness of the poly-Si layer. Therefore, the decline in SS of JL GAA TFTs is due to a decreasing *t*_Si_ and the formation of a crystal-like channel by oxidation. The variation of the SS with temperature $\left(\frac{\partial \mathrm{SS}}{\partial T}\right)$ for JL GAA TFTs is 0.25 mV/dec/K, which is slightly larger than the theoretical value of 0.2 mV/dec/K. The results represent the second term of Equation 1 is small and insensitive to temperature. According to Figure 3b, *I*_off_ is defined as the drain current at *V*_g_ = −1.9 V for JL planar TFTs and at *V*_g_ = −0.2 V for JL GAA TFTs, respectively. Moreover, *I*_off_ can be expressed as follows [9]:

(2) $$I_{\mathrm{off}}=I_{\mathrm{sub}}+I_{\mathrm{leak}}\propto \exp\left(-\frac{qE_{g}}{2\mathrm{kT}}\right),$$

where *I*_sub_ is the subthreshold current, *I*_leak_ is the trap-induced leakage current, and *E*_g_ is the bandgap. The *E*_g_ could be regarded as a constant value for estimation, because $\frac{\partial E_{g}}{\partial T}$ is known to be −0.27 meV/K [10]. Therefore, the *E*_g_ of JL planar and GAA TFTs, as extracted by Equation 2, is around 1.12 and 1.35 eV, respectively. Notably, quantum confinement is observed in JL GAA TFTs, resulting in band-edge shifts (Δ*E*_c_) of the conduction-band and valence-band, thereby increasing the *E*_g_ to reduce the off-state leakage current, as shown in Figure 3c. Figure 3c illustrates the band diagram of the GAA device in off-state with discrete energy levels. The GAA device is simulated by solving 3D quantum-corrected device simulation using the commercial tool, Synopsys Sentaurus Device [11], [12] to obtain accurate numerical results for a nanometer-scale device. These simulation performances are calibrated to experimental data of *I*_d_ – *V*_g_. The Δ*E*_c_ is estimated around 0.23 eV, as extracted from the experimental data in Figure 3b. The theoretical analysis derived from the solution of the Schrödinger equation for the first level in the conduction band as follows [10]:

(3) ΔVth=ΔEcq=h28qme*1Tch2+1W2,

where *m*_e_* is the electron effective mass, *h* is Plank's constant, *T*_ch_ is the channel thickness and *W* is the channel width. The second term in Equation 3, which represents quantum confinement effect in the channel width direction, can be ignored due to *W* > > *T*_ch_. The Δ*V*_th_ of theoretical value is 0.36 eV, which is larger than experimental value of 0.23 eV. The gap would come from the poly-Si channel material.

**Figure 4:**
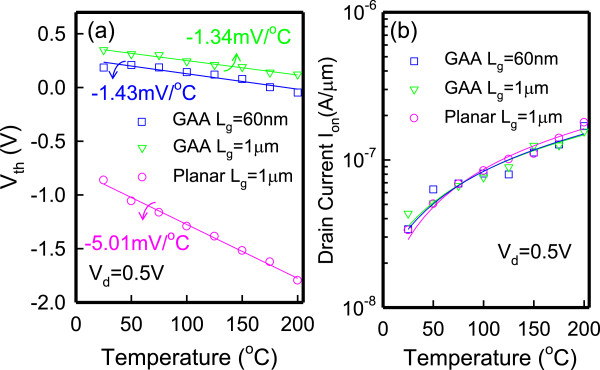
**Impact of temperature dependence on the (a) *V***_**th **_**and (b) on-state currents.** For JL GAA TFTs (*L*_g_ = 1 μm, 60 nm) and JL planar TFTs (*L*_g_ = 1 μm). The V_th_ and I_on_ for JL GAA TFTs are less sensitive to temperature than JL planar TFTs.

Figure 4a presents the measured *V*_th_ as a function of temperature. The *V*_th_ is defined as the gate voltage at *I*_d_ = 10^−9^ A. The temperature coefficients of *V*_th_ are −1.34 and −5.01 mV/°C for GAA and planar JL TFTs, respectively. According to [13], the variation of $\frac{\partial V_{\mathrm{th}}}{\partial T}$ in n-type JL devices can be expressed as follows [13]:

(4) $$\frac{\partial V_{\mathrm{th}}}{\partial T}=\frac{\partial V_{\mathrm{fb}}}{\partial T}‒\left[\frac{q}{\epsilon_{\mathrm{Si}}}{\left(\frac{A}{P}\right)}^{2}+\frac{\mathrm{qA}}{C_{\mathrm{ox}}}\right]\frac{\partial N_{D}}{\partial T}+\frac{1}{q}\frac{\partial \Delta E_{c}}{\partial T},$$

where *V*_fb_ is the flat-band voltage, *C*_ox_ is the gate oxide capacitance per unit length, *A* is the device cross-sectional area and *P* is the gate perimeter. The first term in the right side of Equation 4 is depended on the flat-band voltage variation with temperature. For *N*_D_ = 1 × 10^19^ cm^−3^, the value of $\frac{\partial V_{\mathrm{fb}}}{\partial T}$ is approach to −0.49 mV/°C as the devices in [13], which has a P^+^ polycrystalline silicon gate and the same doping concentration. The second term represents the effect of incomplete ionization. The doped impurities are almost completely ionized at those temperatures higher than room temperature. Thus, the doping concentration variation with the temperature $\left(\frac{\partial N_{D}}{\partial T}\right)$ has a slight dependence on temperature. The third term, depending on the electron effective mass, also has a smaller dependence on *T* than the other terms. The theoretical value of $\frac{\partial V_{\mathrm{th}}}{\partial T}$ is about −0.49 mV/°C; although the $\frac{\partial V_{\mathrm{th}}}{\partial T}$ of −1.34 mV/°C in JL GAA TFTs is larger than theoretical value, but is comparable with current SOI-based JNT ($\frac{\partial V_{\mathrm{th}}}{\partial T}$ approximately −1.63 mV/°C) [7] due to the use of the multi-gate structure and formation of a crystal-like nanosheet channel with fewer traps by oxidation process. Therefore, JL TFTs with the GAA structure and ultra-thin channel shows an excellent immunity to the temperature dependence on *V*_th_ and competes with SOI-based JNT. Figure 4b presents the measured on-current (*I*_on_) as a function of temperature. The *I*_on_ is defined as the drain current at *V*_g_ = 3 V for JL planar TFTs and at *V*_g_ = 6 V for JL GAA TFTs. The JL GAA TFTs show a slightly better *I*_on_ variation with temperature than the planar ones, possibly owing to a smaller $\frac{\partial V_{\mathrm{th}}}{\partial T}$ in JL GAA TFTs.

## Conclusion

This work has presented a high-temperature operation of JL TFTs. The high temperature dependence of JL GAA and planar TFTs is also studied. The variation of parameters such as *V*_th_, *I*_on_, SS, and *I*_off_ are analyzed as well. The variation of the SS with temperature for JL GAA TFTs is close to the ideal value (0.2 mV/dec/K) owing to the ability of the oxidation process to form a nanosheet channel and crystal-like channel. Additionally, *I*_off_ is negligibly small for JL GAA TFTs, owing to quantum confinement effect; its *E*_g_ of 1.35 eV is also extracted. The JL GAA TFTs have a smaller $\frac{\partial V_{\mathrm{th}}}{\partial T}$ than that of JL planar TFTs owing to the GAA structure and ultra-thin channel. Moreover, the measured $\frac{\partial V_{\mathrm{th}}}{\partial T}$ of JL GAA TFTs competes with that of SOI-based JNTs. Therefore, the JL GAA TFTs with a slight variation in temperature performances along with simple fabrication are highly promising for future SOP and system-on-chip SOC applications.

## Competing interests

The authors declare that they have no competing interests.

## Authors’ contributions

YCC and HB handled the experiment and drafted the manuscript. MH made the simulation plot and performed the electrical analysis. NH, JJ, and CS fabricated the samples and carried out the electrical characterization. YCW supervised the work and reviewed the manuscript. All authors read and approved the final manuscript.
